# Biological rationale for the use of DNA methyltransferase inhibitors as new strategy for modulation of tumor response to chemotherapy and radiation

**DOI:** 10.1186/1476-4598-9-305

**Published:** 2010-11-25

**Authors:** Giovanni L Gravina, Claudio Festuccia, Francesco Marampon, Vladimir M Popov, Richard G Pestell, Bianca M Zani, Vincenzo Tombolini

**Affiliations:** 1 Department of Experimental Medicine, Division of Radiation Oncology, S. Salvatore Hospital, L'Aquila, University of L'Aquila, Medical School, L'Aquila 67100, Italy; 2 Department of Experimental Medicine, Laboratory of Radiobiology, University of L'Aquila, Medical School, L'Aquila 67100, Italy; 3 Department of Cancer Biology and Medical Oncology, Kimmel Cancer Center, Thomas Jefferson University, Philadelphia, PA 19107, USA

## Abstract

Epigenetic modifications play a key role in the patho-physiology of many tumors and the current use of agents targeting epigenetic changes has become a topic of intense interest in cancer research. DNA methyltransferase (DNMT) inhibitors represent a promising class of epigenetic modulators. Research performed yielded promising anti-tumorigenic activity for these agents *in vitro *and *in vivo *against a variety of hematologic and solid tumors. These epigenetic modulators cause cell cycle and growth arrest, differentiation and apoptosis. Rationale for combining these agents with cytotoxic therapy or radiation is straightforward since the use of DNMT inhibitor offers greatly improved access for cytotoxic agents or radiation for targeting DNA-protein complex. The positive results obtained with these combined approaches in preclinical cancer models demonstrate the potential impact DNMT inhibitors may have in treatments of different cancer types. Therefore, as the emerging interest in use of DNMT inhibitors as a potential chemo- or radiation sensitizers is constantly increasing, further clinical investigations are inevitable in order to finalize and confirm the consistency of current observations.

The present article will provide a brief review of the biological significance and rationale for the clinical potential of DNMT inhibitors in combination with other chemotherapeutics or ionizing radiation. The molecular basis and mechanisms of action for these combined treatments will be discussed herein.

## 

A significant number of tumors are classified as poorly or non-responsive to therapeutic drugs or radiotherapy. Increasing the chemotherapeutic dosage or radiation dose not only fails in improving the therapeutic response, but it also contributes to the development of side effects and resistance to therapy. An ideal strategy would consist of the identification of anticancer agents able to act synergistically with standard treatments such as radiotherapy and chemotherapy, which would result in triggering the cell death preferentially in tumor cells. Many patients with neoplastic diseases exhibit hypermethylation of cytosine residues in gene promoters which induce silencing of key tumor suppressor genes. Since methylation of CpG islands occurs infrequently in normal cells, the modulation of this post-translational modification may provide a selective tumor-specific therapeutic target.

The packaging of DNA is critical for many DNA metabolic processes including transcription, replication and DNA repair. DNA is normally tightly wrapped around histone octamers to form nucleosomes. These primary elements have been traditionally thought as stable DNA packaging units. However, it is now evident that they are dynamic structures that can be altered by different molecular processes [1-3]. These include (i) incorporation of histone variants, which are thought to have specialized functions [4], (ii) replacement, repositioning or, in certain cases, the removal of nucleosomes by chromatin remodeling complexes, and finally (iii) post-translational modifications.

Post-translational modifications include (i) lysine acetylation and deacetylation, (ii) methylation, (iii) serine phosphorylation and ubiquination and (iv) lysine sumoylation. These modifications play a major role in modeling higher-order chromatin conformation and modifying the DNA accessibility to transcription factors [5,6]. Therefore, such changes are not strictly "genetic," even though the specific chromatin patterns are usually inherited by daughter cells during replication.

In cancer, epigenetic silencing through methylation occurs just as frequently as mutations or deletions and leads to aberrant silencing of genes with tumor-suppressor functions [2,3].

Among the post-translational processes, DNA methylation is one of the most extensively characterized epigenetic modifications and its biological role is to maintain DNA transcriptionally quiescent, resulting in gene silencing (Figure 1) [7-9]. This process is dependent upon the action of DNA methyltransferases (DNMTs), enzymes that catalyze the addition of methyl groups to the 5' carbon of the cytosine residues (Figure 1) [7]. Several isoforms of DNMTs are present in normal cells as well as in tumor cells [9-11]. Existing evidence indicates that DNMT1 appears to be responsible for maintenance of established patterns of methylated DNA, while DNMT-3a and -3b seem to mediate *de novo *DNA methylation patterns [9-11]. Interestingly DNMT1 alone is not sufficient for maintenance of abnormal gene hypermethylation but the cooperation with DNMT3b must occur for this function [12-14]. In the last years many different DNMT inhibitors have been developed (Table 1) and multiple molecular mechanisms by which DNMT inhibitors induce anti-cancer effects have been identified. These mechanisms are partially mediated by the hypomethylation of DNA with cytotoxic effects documented at higher concentrations [8,15]. The net effect is the modulation of specific genes involved in cellular processes such as apoptosis, cytostasis, differentiation and tumor angiogenesis [8,15]. Therefore, it is not surprising that DNMT inhibitors are emerging as promising class of drugs in cancer treatment, especially in combination with other agents or with other treatments like radiotherapy. Even though some DNMT inhibitors have entered into clinical trials, we currently have limited understanding of their precise mechanisms of action, especially when combined with other available treatments.

**Figure 1:**
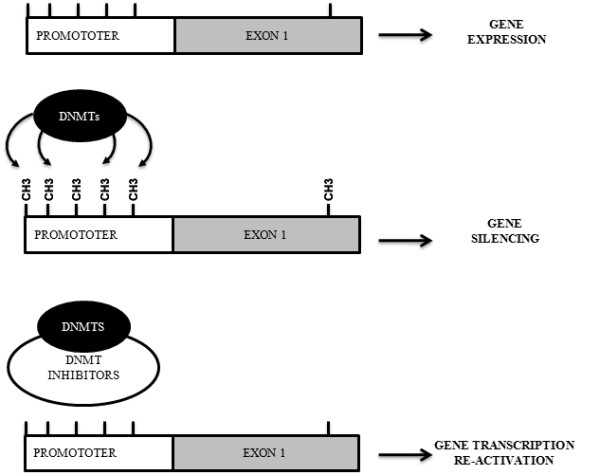
**Epigenetic modulation of gene expression by post-translational DNA methylation**. Transcriptionally inactive chromatin is characterized by the presence of methylated cytosines within CpG dinucleotides (CH3), which is sustained by DNA methyltransferases (DNMTs).

**Table 1:** Overview of some DNMT inhibitors with their mechanisms of action

Name	Chemical nature	Mechanism of action
**Azacitidine**	Ribonucleoside analogue	This drug is a ribonucleoside analogue and it binds to RNA and DNA. This molecule interrupts mRNA translation and when incorporated into DNA inhibits methylation by trapping DNMTs. At relatively higher concentrations this drug results in the formation of high levels of enzyme-DNA adducts.

**Decitabine**	Deoxyribonucleoside analogue	This drug is a deoxyribonucleoside analogue. For this reason, this molecule does not bind to RNA but only to DNA. When incorporated into DNA inhibits methylation by trapping DNMTs resulting in the reduced methylation of cytosines in DNA synthesized after drug treatment. When used at relatively high concentrations this drug results in the formation of high levels of enzyme-DNA adducts,

**Zebularine**	Deoxyribonucleoside analogue	This drug is a deoxyribonucleoside analogue. For this reason, this molecule does not bind to RNA but only to DNA. When incorporated into DNA inhibits methylation by trapping DNMTs resulting in the reduced methylation of cytosines in DNA synthesized after drug treatment. When used at relatively high concentrations this drug results in the formation of high levels of enzyme-DNA adducts,

**(−)-epigallocatechin-3-gallate**	Non-nucleoside analogue	

**MG98**	Non-nucleoside analogue	This antisense oligonucleotide targets the 3 UTR of DNMT1 causing a methylation decrease in cell lines and animal models

**RG108**	Non-nucleoside analogue	This small molecule is not incorporated into DNA but i bind to the catalytic site of DNMTs causing inhibition of DNA methylation

**Procainamide**	Non-nucleoside analogue	This molecule reduces DNMT1's affinity for both DNA and *S*-adenosyl-methionine causing a decrease in DNA methylation

The present article will provide a brief review of the biological significance and scientific rationale for the clinical potential of DNMT inhibitors in combination with chemotherapy or radiotherapy.

## Combined therapy: Published Experience, Ongoing Studies and Future directions

The goal of combining different treatments in the management of cancer is to increase and prolong the response rate as well as to decrease the toxicity associated with each treatment. Two different strategies can be utilized to achieve these objectives. Treatments may be combined based on the absence of overlapping or synergistic toxicities leading to empiric combinations. A more sound approach is based upon the combination of treatments with known convergent molecular mechanisms.

It is well known that epigenetic abnormalities in cancer affect a plethora of genes involved in key cellular pathways including cell cycle control, apoptosis, immune recognition, angiogenesis tumor invasion and metastasis. Consistent with the functional diversification of epigenetic alterations, epigenetic drugs are characterized by pleiotropic effects which affect key aspects of tumor biology leading to an overall impairment of the neoplastic potential of tumor cells. These are the reasons that constitute the rationale for the proposed usage of DNMT inhibitors as anticancer agents, alone or in combination with other treatments.

### DNMT inhibitors and chemotherapy

Despite the promising anticancer activity in haematological malignancies [16], early clinical trials showed that DNMT inhibitors have low anticancer activity and significant toxicity as single agent in solid tumors. Recent studies, however, suggest that low concentrations of DNMT inhibitors such as 5-Aza and decitabine may act synergistically when combined with chemotherapy and contribute to overcoming intrinsic or acquired chemoresistance [17-19]. These properties are considered clinically significant as the resistance of tumor cells to cytotoxic agents remains the major obstacle in chemotherapeutic-based treatments. The mechanisms underlying chemoresistance remain in some measure elusive even though multifactorial mechanisms, including epigenetic modifications may drive this mechanism [20-25]. Therefore, any effort to overcome multi-drug resistance represents the primary goal in cancer research. Based on the chemical mechanisms, DNMT inhibitors act through different mechanisms. Among the different mechanisms postulated, alterations in differentiation, changes in apoptosis, and induction of a beneficial immune response are considered of main importance [19]. Finally, the induction of DNA damage due to the formation of irreversible covalent enzyme-DNA adducts has also been taken into consideration.

### Cell signaling

**Figure 2:**
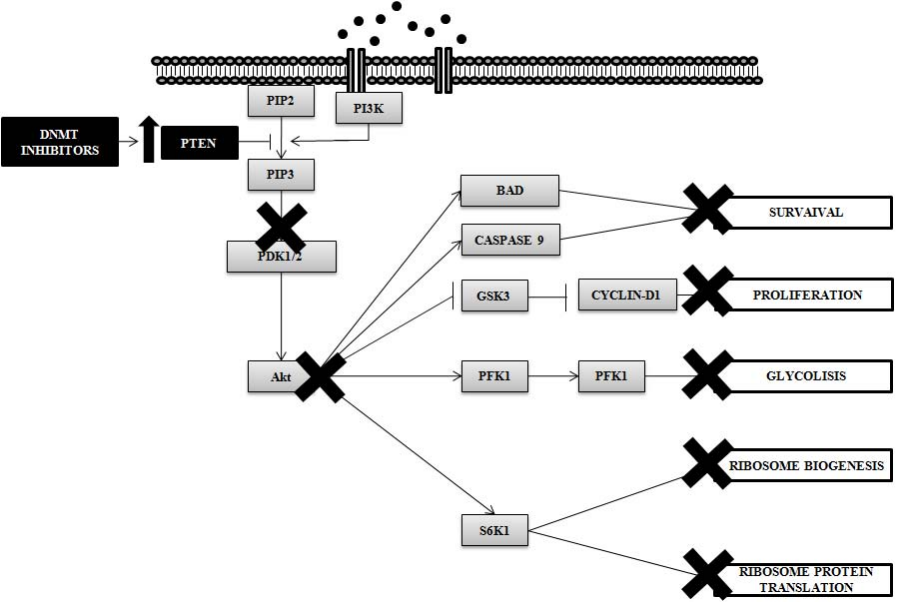
**DNMT inhibitors and PTEN/PI3K/Akt pathway**. PTEN/PI3K/Akt pathway physiologically plays a key role in the control of many processes essential for the cellular life. The tumor suppressor PTEN negatively controls the PI3K/Akt pathway and its epigenetic loss, frequent in cancer cells, leads to the aberrant pathway activation. DNMT inhibitors restore the PTEN expression by epigenetic mechanisms.

Cell signaling is a complex system of communication that coordinates basic cellular activities. Cells perceive and correctly respond to microenvironment via this complex system engaging in cellular processes such as, tissue repair, immunity, as well as normal tissue homeostasis. Errors in cell signaling are involved in the development as well as in the progression of cancer and aberrant DNMT activity has been involved in these processes. PTEN (phosphatase and tensin homolog deleted on chromosome ten) is a tumor suppressor gene and its functional loss has been documented in bladder cancer, glioblastoma, melanoma and cancers of the prostate, breast, lung and thyroid [26-30]. This tumor suppressor gene controls PI3K by preventing the activation of PDK-1 and Akt (Figure 2). The functional loss of PTEN is higher than that attributable to LOH of chromosome 10q and post-translational mechanisms, including hypermethylation, explain the other part of this phenomenon [31-34]. Evidence indicates that 5-Aza is a chemosensitizer in prostate cancer [35] and its property seems mediated by PTEN. After infection with a recombinant adenovirus containing wild-type PTEN, bladder tumor cells acquire greater chemosensitivity to the cytotoxic effect of doxorubicin [36-39]. The chemosensitivity induced by PTEN is partially mediated by PI3K and Akt/PKB [40]. Other evidence, in a different experimental model, suggests that the transfection of TC-32 Ewing sarcoma cells with Akt/PKB inhibits doxorubicin-induced apoptosis suggesting that PTEN increases doxorubicin cytotoxicity through the PI3K signaling pathway. Additional indication of chemosensitizing properties of PTEN derived from data obtained in endometrial cancer cells [41]. In this system, PTEN significantly enhanced chemosensitivity to doxorubicin. This effect was associated with the levels of phospho-Akt/PKB and phospho-Bad (Ser-136), which were reduced in the PTEN expressing clones. Results from other studies performed on brain tumors clearly show that decreasing activity of the PI3K/Akt pathway in tumor cells with mutant PTEN may contribute to the increased sensitivity to chemotherapy [42]. Down-regulating the Akt pathway by inducing PTEN also increases the sensitivity of glioblastoma cells to temozolomide.

### DNA base damage

Some kind of acquired or intrinsic chemoresistance may be epigenetic in nature and operate at DNA mismatch repair level [43-48]. Interestingly, suppression of a DNA mismatch repair mechanism seems [49] to act in concert with other independent DNA mismatch repair machineries [50,51], resulting in drug resistance and genetic instability [52]. The suppression of DNA mismatch repair mechanism can occur at epigenetic level and in the absence of heritable inactivating mutations. Demethylating agents have been shown to reverse drug resistance to alkylating agents in some preclinical models of ovarian and colorectal cancer [53]. This process may happen through up-regulation of MLH1, and is mediated by 5-Aza treatment, which sensitizes tumor cells to cisplatin. However, 5-Aza treatment does not sensitize MLH1 mutant cells to cisplatin, indicating that MLH1 gene reactivation is required for the sensitization [53,54]. If MLH1 reactivation is required for the sensitization to cisplatin, several concerns still exist about the value of epigenetic modulation of DNA repair genes in inducing chemosensitization. It is well known that MGMT, another DNA repair mediator, is frequently epigenetically silenced [55]. Consistent with its role in protecting the genome from G to A transitions, induced by alkylating agents, MGMT inactivation through its promoter hypermethylation has been associated with G to A mutations in k-ras and p53 genes in colorectal cancer [56,57]. Even though DNMT inhibitors, such as 5-Aza and decitabine, have proven effective in re-expressing MGMT in cancer cells, the clinical advantage of the restored MGMT expression is doubtful [58-60] since tumors with the unmethylated promoter of MGMT gene appear significantly less susceptible to the cytotoxic effects of alkylating drugs [56,57,60].

Although the biological effects of DNMT inhibitors on methylation and demethylation status of DNA mismatch repair genes have been extensively studied, DNA damage-related sequelae of these agents is still not fully understood. DNA Double Strand Breaks (DSBs) are the most cytotoxic DNA lesions. One-ended DSBs can be formed via collapse of a replication fork at the site of a blocking DNA lesion [61-64]. Given that DNMT inhibitors, as well as some chemotherapeutics, create irreversible covalent DNA-enzyme adducts, the convergence of these phenomena may be one possible mechanism by which these two agents synergize and induce cytotoxicity [65,66]. Several reports indicate that 5-Aza, decitabine and zebularine induce DSB responses followed by induced apoptosis. These responses may be mediated via ATR or ATM, which are two key mediators promoting DNA DSB response signalling [67-69]. Given that the DSB responses induced by 5-Aza and doxorubicin engage in distinct signaling pathways, the combination of these two agents may cooperate and synergistically induce cell death [70]. The induction of DNA damage response by Chk2 and p53 phosphorylation could be another mechanism by which DNMT inhibitors induce DNA damage-related sequelae and cooperate with chemotherapeutics [71-75]. In this regard, some studies revealed that decitabine may be cytotoxic against both p53 wild-type and p53 mutant containing tumor cells, suggesting that p53 function is not always required in order to mediate the apoptotic process of DNMT inhibitors. Finally, it has been demonstrated that these agents can induce DNA damage in a dose-dependent manner, while the degree and the kind of DNA damage induced parallels the amount of incorporated DNMT inhibitor [76]. This may be an important cooperative mechanism since when the cytotoxic effect of DNMT inhibitors takes place in close proximity of a single- or double-strand break induced by chemotherapeutics the damage may be lethal.

### Apoptosis

Defects in apoptotic pathways promote chemoresistance. DNMT inhibitors are known to potentiate apoptotic processes through different pathways. Evidence suggests that the induction of TRAIL by decitabine is critical for sensitizing breast cancer cells to Adriamycin. The silencing of TRAIL decreases caspase activation and abrogates chemosensitization mediated by decitabine. Several mechanisms by which DNMT inhibitors induce TRAIL have been postulated. One of the possible mechanisms is the activation of TRAIL gene expression [77,78]. Additional evidence suggests that the induction of TRIAL by decitabine is mediated by the increase in the half-life of TRAIL protein [78] or by the induction of TRAIL via Akt. It is known that the PI3K inhibitor wortmannin can induce TRAIL [79], and that the overexpression of the active form of Akt can abolish TRAIL induction mediated by wortmannin. In agreement with this evidence, Akt, working as a negative modulator of TRAIL, is modulated by 5-Aza resulting in a decrease of phosphorylated Akt and enhanced TRAIL expression.

If TRAIL plays a key role in the apoptotic process mediated by DNMT inhibitors, other investigators suggest that the methylation in the promoter region of caspase 8 and caspase 9 is another well known mechanism by which tumors acquire chemoresistance. Mechanistic studies indicate that decitabine induces caspase-8 and caspase-9 and sensitizes tumor cells to TRAIL, etoposide, cisplatin [80,81] and paclitaxel [82].

Overexpression of the Activator protein 2α (AP-2α) is another mechanism through which DNMT inhibitors may induce apoptosis and influence chemosensitivity. AP-2α is a sequence-specific DNA-binding transcription factor that is required for regulation of many genes involved in many biological functions [83-85]. Growth inhibitory activity of AP-2α is mediated through direct interaction with p53 [86] and the overexpression of this transcription factor induces cell cycle arrest and apoptosis [87,88]. Epigenetic targeting of AP-2α inhibits tumor proliferation and increases tumor cell death [89]. This acquires a meaningful clinical significance considering that 75% of invasive breast tumors have epigenetically silenced AP-2α. Therefore, the use of DNMT inhibitors may provide the unique opportunity for modifying the chemosensitivity of breast cancer containing hypermethylated and silenced AP-2α [90-95].

### Oxidative stress

Agents inducing oxidative stress determine cellular damage by reactive oxygen intermediates (ROI). Small amounts of ROI may act as signalling molecules but if ROI production exceeds the endogenous intracellular antioxidative capacities [96,97] an oxidative stress occurs [98] resulting in cell death. Increased ROI levels contribute to the development of chemoresistance and growing evidence supports a role of epigenetic processes in ROS-induced generation of oxidative stress [99-105]. The proto-oncogene AP-1 plays a central role in the control of cellular response to oxidative stress [106-109]. It modulates the expression of target genes involved in protective and/or reparative cellular responses to the damaging effects of oxidative stress [110-113]. Experimental data suggest that H2O2 stress-resistant tumor cells have increased AP-1 DNA-binding activity and are resistant to the damaging effects of chemotherapeutic agents [114]. The inhibition of the AP-1 complex reverses the multimodality resistance phenotype (MMRP) in response to oxidative stress through the inhibition of Fos activity [114]. Other studies have expanded these observations showing that DNMT1, a downstream target of Fos, is upregulated in chemoresistant tumor cells [114]. These results indicate that the epigenome may play a critical role to oxidative stress and highlights a potential role of DNMT1 activity abrogation as a potential molecular target in chemoresistant tumor cells. This evidence has been further confirmed showing that the selective silencing of DNMT1 and DNMT3b greatly reduces the chemoresistance of tumor cells overexpressing DNMTs isoenzymes [115].

### DNMT inhibitors and radiotherapy

**Figure 3:**
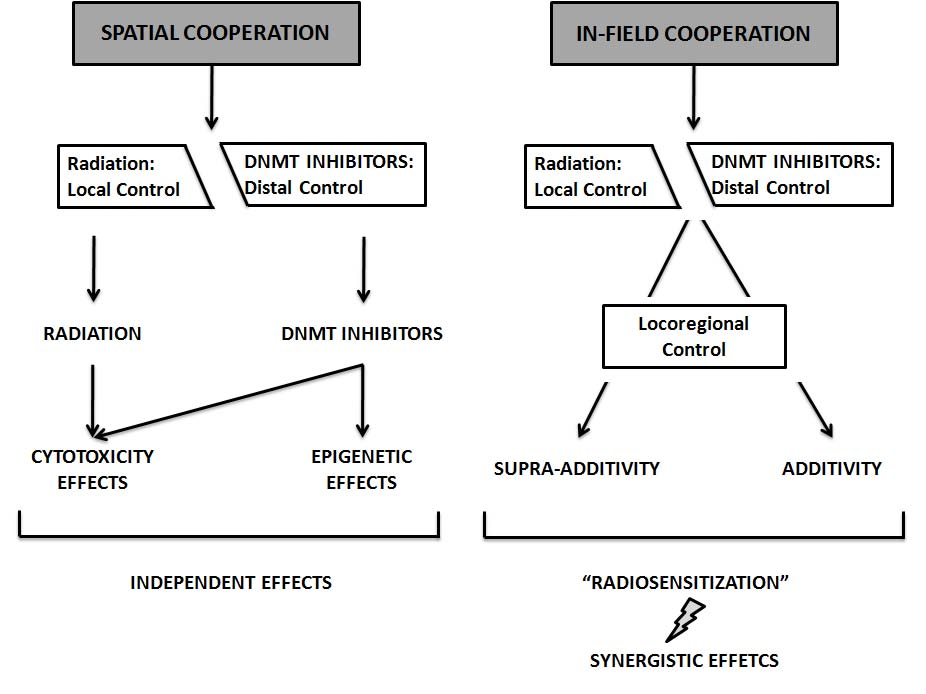
**Rationale for combining DNMT inhibitors and radiation therapy**. Spatial and in-field cooperation are the two modalities of cooperation mechanisms between DNMT and ionizing radiation.

The therapeutic index of radiotherapy can be improved by chemical agents that sensitize cancer cells to the toxic effects of ionizing radiation. Radiotherapy and systemic agents may interact through two main modalities (Figure 3). In the first modality, radiotherapy acts locoregionally and systemic agents act against micro-metastases without interaction between the treatments. In the second modality radiotherapy and systemic agents interact within the radiation field increasing tumor cell death.

Sparse biological data indicate that DNMT inhibitors may act as radiosensitizers. The meaningful advantage of DNMT inhibitors as radiosensitizers is that these agents can induce radiosensitization at concentrations several times lower than typical plasma levels obtained when used as single agents [116]. Major interactions between DNMT inhibitors and radiotherapy may be postulated. For clearer understanding, the potential biological mechanisms of cooperation between DNMT inhibitors and radiotherapy will be discussed separately.

#### DNA repair

**Figure 4:**
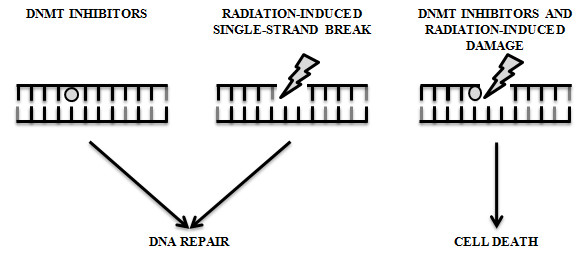
**Cooperative cytotoxic mechanism between DNMT inhibitors and radiation**. Ionizing radiation induces DNA base damage, single-strand breaks, and double-strand breaks (DSBs). All of these errors can be rapidly repaired except for DSBs, which if not repaired are considered lethal. The cytotoxic effect of DNMT inhibitors in close proximity to a radiation-induced single-strand break can act synergistically to make the defect significantly more difficult to repair, consequently resulting in the induction of cellular death.

Radiation therapy induces DNA base damage, single-strand breaks, and double-strand breaks (DSBs). These structural damages are repairable, except for DSBs, which are considered lethal [116]. 5-Aza, decitabine and zebularine lead to protein-DNA adducts and when the cytotoxic effect takes place in close proximity to a radiation-induced single-strand break, the damage may be significantly more difficult to repair (Figure 4). The DNA cytosine-C5 methyltransferase (MTase) acts on a cytosine residue through its recognition sequence by covalently binding to C6, and then transferring the methyl group from S-adenosylmethionine to C5. The covalent protein-DNA linkage is then reversed and the enzyme dissociates from the DNA. In this context, 5-Aza, decitabine and zebularine substitution at the target cytosine interferes with the reaction cycle, which results in long-lived or irreversible MTase-DNA adducts [117-121]. The cytotoxic mechanism of DNMT inhibitors has been documented in *in vivo *models. Results from these studies suggest that (i) the formation of protein-DNA adducts mediate decitabine cytotoxicity [122], (ii) the cytotoxicity levels correlate positively with MTase levels [123], and (iii) decitabine induce p53 DNA damage response by MTase-DNA adducts [124-127].

#### Cell cycle

**Figure 5:**
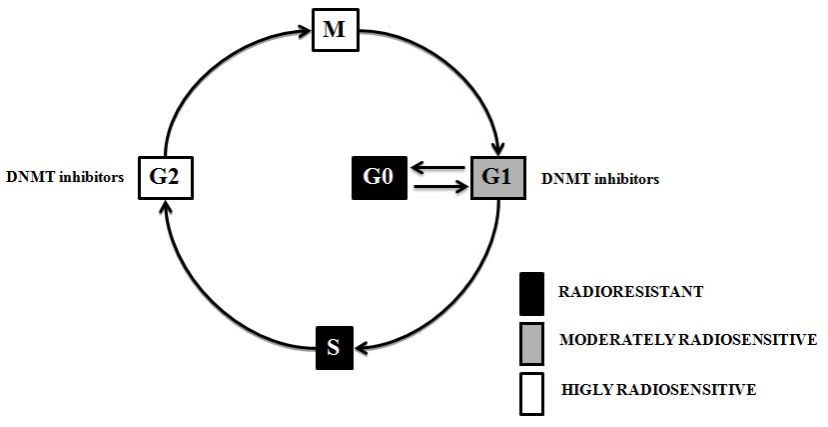
**Cell cycle, DNMT inhibitors and radiosensitivity**. The radiosensitivity of cells is dependent on the phase of the cell cycle. Cells in the S phase are the most radio resistant, and cells in the G2-M phase of the cell cycle are the most radiosensitive. DNMT inhibitors synchronize with the cell cycle of tumor cells increasing the efficacy of subsequent radiotherapy.

The radiosensitivity of tumor cells is dependent on the phase of the cell cycle. Cells in the S-phase are the most radioresistant, whereas cells in the G2-M phase are the most radiosensitive. Evidence indicates that DNMT inhibitors synchronize tumor cells preferentially in the G1 or G2/M phase of the cell cycle increasing the efficacy of radiotherapy (Figure 5). In this way, if administered concurrently to radiotherapy, the inhibitors may cooperate to produce additive or synergistic antitumor effects. *Qui *and co-workers demonstrated that low concentrations of decitabine synchronize gastric tumor cells in the G2/M phase of the cell cycle and induce radiosensitization [116]. The radiosensitizing effect of this inhibitor seems to be partly mediated by *p53*, *RASSF1*, and *DAPK *[116]. *RASSF1 and DAPK *modulate multiple apoptotic and cell-cycle checkpoint pathways [128,129] and the loss of *RASSF1 *and *DAPK *expression is documented in a wide range of human tumors as a result of silencing, primarily from promoter hypermethylation [130]. Therefore, the epigenetic modulation of *RASSF1 and DAPK *shines some light on the synergism between DNMT inhibitors and radiotherapy in terms of apoptotic signaling modulation.

#### Angiogenesis

Induction of anti-angiogenic activity in radiotherapy as a result of combined treatments with DNMT inhibitors is backed by a clear rationale. Common in many cancers hypoxia has been indicated as a marker of aggressive clinical behaviour, poor prognosis and unsatisfying radiation response. Therefore, any compound able to increase perfusion and oxygenation reduces the radioresistant hypoxic areas counteracting the onset of hypoxic and radioresistant clones.

Experimental data suggest that many key modulators of angiogenesis are under epigenetic control. The promoter of von Hippel-Lindau (VHL) tumor suppressor gene is hypermethylated and its absence leads to a failure in degradation of hypoxia inducible factor (HIF)-1 whose accumulation favors tumor angiogenesis [131]. This aberrant pathway has been successfully inactivated by decitabine, which in addition to restoring VHL expression, down-regulates the vascular endothelial growth factor (VEGF), the glucose transporter (GLUT)-1 [132] and the thrombospondin-1 [133]. Radiotherapy has been shown to kill proliferating endothelial cells. Therefore, any drug that acts on endothelial cells induces a significant increase in the cytotoxic effect of radiotherapy decreasing the levels of angiogenesis. DNMT inhibitors act directly on activated endothelial cells and inhibit angiogenesis *in vitro *and *in vivo *[132]. Decitabine and its analogue zebularine exhibit significant angiostatic activity. This is accompanied by a significant effect on the expression levels of angiogenesis inhibiting genes (TSP1, JUNB, and IGFBP3). TSP1 is known to block endothelial cell migration and to induce apoptosis. JUNB negatively regulates cell growth by activating p16INK4A and decreasing cyclin D1 expression [132], while IGFBP3, a key regulator of cell growth and apoptosis, inhibits VEGF-mediated HUVEC proliferation [133] and angiogenesis [133]. Re-expression of these growth-inhibiting genes by DNMT inhibitors, in activated endothelial cells, may contribute to a decrease in angiogenesis and improvement in intrinsic radiosensitivity.

#### Apoptosis

Apoptosis is a well known mechanism through which antitumor agents induce radiosensitization. DNMT inhibitors sensitize tumor cells to apoptosis either by restoring the defective expression of apoptotic effector proteins, or by re-establishing the expression of signal transducing/mediators of the apoptotic signals. 5-Aza restores the expression of DAPK1 in bladder carcinoma and B-cell lines [134] and the re-expression of DAPK1 in Burkitt's lymphoma cell lines restores the susceptibility to IFN-α triggered apoptosis [135]. Similarly, DNMT inhibitors sensitize NSCLC cells to TRAIL-induced apoptosis by inducing DAPK1 expression [136]. Besides DAPK1, 5-Aza and DNMT1 antisense oligonucleotides are able to restore the sensitivity of cancer cells to IFN-triggered apoptosis despite the re-expression of the pro-apoptotic gene RASSF1A and XAF1 which are frequently silenced by epigenetic mechanisms [137,138]. Caspases are not spared from epigenetic inactivation during tumor transformation. Hypermethylation of caspase-8 promoter leads to its either reduced expression or complete absence in neoplastic cells resulting in their resistance to death receptor and drug-induced apoptosis. However, 5-Aza has proven to be effective in re-establishing caspase-8 expression in cancer cells, restoring their sensitivity to TRAIL-, anti-FAS-, and drug-triggered apoptosis [35,139-141].

#### Cell signaling

DNMT inhibitors are modulators of gene expression and may increase the expression levels of many key genes, specifically the ones involved in the radiosensitizing processes. NF-κB is capable of activating a number of genes involved in stress response, inflammation, and apoptosis. Loss or inhibition of NF-κB activation leads to radio-sensitization [142-144]. The elevated basal NF-κB activity in certain cancers has been linked with tumor resistance to chemotherapy and radiation [145]. Together with the assumption that NF-κB is capable of regulating more than 150 effector genes, this transcription factor plays a key role in tumor radioadaptive resistance under fractional ionizing radiation. 5-Aza can rapidly induce inhibition of NF-kB [146]. This effect may be achieved via down-regulation of pro-survival and anti-apoptotic (IL-6, IL-6Ra, Bcl-XL) mediators or abrogating drug-induced NF-kB stress responses [147,148]. PARP-1 represents the essential transcriptional co-regulator implicated in radiation-induced NF-κB, AP-1, Oct1, and HIF-1α activation [149]. Experimental results demonstrate that the ATM gene is a target for silencing through aberrant methylation of its proximal promoter region [150,151]. This epigenetic event can result in a decreased expression of ATM, resulting in a radioresistant phenotype consistent with reduced ATM function. DNMT inhibitors, positively affecting the levels of ATM, increase radiosensitivity in human colorectal tumor cell lines. In this regard, the moderate radiosensitivity displayed by HCT-116 cells can be increased by 5-azacitidine treatment, correlating with ATM levels [151].

### Epigenetic control of oncogenes: implication for standard treatments

Global genomic hypomethylation has been documented in most solid tumors [152-154]. Evidence suggests that this post-translational mechanism supports tumor development [155]. In solid human tumors, a correlation between global genomic hypomethylation and advanced tumor stage has been established [154].

Methylation has been primarily considered as a mechanism for tumor suppressor genes silencing and genome profiling approaches have identified several putative tumor suppressor genes silenced by promoter hypermethylation. So far, unmasked expression of putative oncogenes has been sporadically reported [156].

Although c-*myc *was among the very earliest oncogenes identified and the subject of intense study, it has nonetheless proven to be an enduring enigma. Results to date suggest that Myc-Max influences cell growth and proliferation through direct activation of genes involved in DNA synthesis, RNA metabolism and cell-cycle progression [157]. Early studies showed that c-myc is under epigenetic control and its functional silencing sensitizes cancer cells to chemotherapy and radiotherapy [158-160]. These sensitizing effects of c-Myc were primarily achieved by inhibiting MLH1 and MSH2 mismatch repair proteins [161]. Evidence suggests that decitabine is unable to modify the expression of c-myc in gastric cancer [161]. Other evidence, however, suggest that several proto-oncogenes, whose promoters are under epigenetic control, may be down-regulated rather than up-regulated after treatment with epi-drugs [162]. Microarray data revealed that the treatment of myeloma multiple cells by decitabine and TSA resulted in down-regulation of several proto-oncogenes such as members of myc family [162]. Of note, the down-regulation of these genes was more a response to TSA and decitabine/TSA than to decitabine alone. The biological rationale for this surprising phenomenon is not well known although this effect may be explained either by a direct inhibitory action of decitabine and TSA or by an indirect down-regulation by decitabine and TSA affected genes [162].

Therefore, these conflicting data may have important therapeutic implications since demethylation-based therapy can cause unintended effects. It may be possible that in certain tissues and under selective biological conditions epi-drugs may result in either up- or down-regulation of proto-oncogenes [163]. These concerns may explain either some of the side effects or the unsuccessful results documented upon demethylation-based therapy in solid tumors.

### Response of normal tissues to DNMT inhibitors

The use of DNMT inhibitors raises questions regarding their potential to epigenetically affect non-cancerous cells. Therefore, an important issue is a need for a more complete understanding of the potential benefits and limitation of DNA methylation as a human cancer drug target. Conflicting evidence exist in literature regarding the effect of DNMT inhibitors on normal cells. Even though well known toxicity profile has been documented for DNMT inhibitors, especially for nucleoside analogues such as 5-Aza and decitabine in the clinical setting (Table 2), many doubts exist about their long-term safety as well as about their mutagenic and carcinogenic potential [164]. Some evidence indicate that intraperitoneal injection of 5-Aza at doses ranging from 2.0 to 2.2 mg/kg for 50-52 weeks in murine models increased the incidence of malignant tumors of hematopoietic and lymphoreticular system as well as of lung, mammary glands and skin [165]. The mutagenic potential of 5-Aza [165] and decitabine [166] was tested *in vitro *and *in vivo *systems. Both analogues increased mutation frequency in L5178Y mouse lymphoma cells, and mutations were produced in an *Escherichia coli *lac-I transgene in colonic DNA of decitabine-treated mice [166]. Decitabine, moreover, resulted in chromosomal rearrangements in larvae of fruit flies. The effect of decitabine and 5-Aza on postnatal development and reproductive capacity was evaluated in murine models. Administration of these inhibitors in male mice resulted in decreased fertility and loss of offspring during subsequent embryonic and postnatal development. Decreased weight of the testes and epididymides with reduced sperm counts were also detected. Body weights of males and females exposed in utero to decitabine were significantly reduced at all postnatal time points. No consistent effect on fertility was seen when female mice exposed in utero.

**Table 2:** Clinical toxicity

	5-Azacitidine	Decitabine
General	Pyrexia, fatigue, weakness, rigors, pain in limb, back pain, contusion, dizziness, erythema, chest pain, epistaxis, myalgia, decreased weight, abdominal pain, aggravated fatigue, abdominal tenderness, insomnia, malaise, pain, upper abdominal pain, night sweats, lethargy, peripheral swelling, transfusion reaction, abdominal distension, syncope, chest wall pain, hypoesthesia, post procedural pain, general physical health deterioration, systemic inflammatory response.	Pyrexia, peripheral edema, rigors, edema, pain, lethargy, tenderness, fall, chest discomfort, intermittent pyrexia, malaise, crepitations.

Local	Injection site erythema, injection site pain, injection site bruising, injection site reaction, injection site pruritus, injection site granuloma, injection site pigmentation changes, injection site swelling.	Erythema, catheter site pain, and injection site swelling.

Cardiovascular		Cardiac murmur, hypotension, pulmonary edema.

Respiratory	cough, dyspnea, nasopharyngitis, exertional dyspnea, productive cough, pneumonia, lung crackles, rhinorrhea, rales, wheezing, decreased breath sounds, pleural effusion, postnasal drip, rhonchi, nasal congestion, atelectasis, exacerbated dyspnea, sinusitis, hemoptysis, lung infiltration, pneumonitis, respiratory distress	cough, pharyngitis, lung crackles, decreased breath sounds, hypoxia, rales, postnasal drip.

Musculoskeletal	arthralgia, muscle cramps, aggravated bone pain, muscle weakness, and neck pain.	arthralgia, limb pain, back pain, chest wall pain, musculoskeletal discomfort, myalgia

Hematologic	anemia, thrombocytopenia, leukopenia, neutropenia, febrile neutropenia, hypokalemia, post procedural hemorrhage, aggravated anemia, agranulocytosis, bone marrow depression, bone marrow failure, pancytopenia, and splenomegaly.	neutropenia, thrombocytopenia, anemia, febrile neutropenia, leukopenia, lymphadenopathy, and thrombocythemia.

Gastrointestinal	nausea, vomiting, diarrhea, constipation, anorexia, pharyngitis, appetite decreased, gengival bleeding, oral mucosal petechiae, stomatitis, dyspepsia, hemorrhoids, loose stools, dysphagia, mouth hemorrhage, tongue ulceration, diverticulitis, gastrointestinal hemorrhage, melena, perirectal abscess	nausea, constipation, diarrhea, vomiting, abdominal pain, oral mucosal petechiae, stomatitis, dyspepsia, ascites, gingival bleeding, hemorrhoids, loose stools, tongue ulceration, dysphagia, oral soft tissue disorder, lip ulceration, abdominal distension, abdominal pain upper, gastroesophageal reflux disease, glossodynia.

Dermatologic	ecchymosis, petechiae, skin lesions, rash, pruritus, increased sweating, urticaria, dry skin, skin nodule, pyoderma gangrenosum, pruritic rash, and skin induration.	ecchymosis, rash, erythema, skin lesion, pruritus, alopecia, urticaria, and facial swelling.

Immunologic		Infections and infestations such as pneumonia, cellulitis, candidal infection, catheter related infection, urinary tract infection, staphylococcal infection, oral candidiasis, sinusitis, bacteremia.

Nervous System	headache, convulsions, intracranial hemorrhage.	headache, dizziness, hypoesthesia, insomnia, confusional state, anxiety.

Others	pallor, pitting edema, lymphadenopathy, hematoma, cellulitis, infections and infestations including herpes simplex, limb abscess, bacterial infection, blastomycosis, injection site infection, Klebsiella sepsis, streptococcal pharyngitis, Klebsiella pneumonia, sepsis, Staphylococcal bacteremia, Staphylococcal infection, neutropenic sepsis, septic shock, toxoplasmosis, genitourinary infection, hematuria.	vascular disorders such as petechiae, pallor, hematoma, increased blood alkaline phosphatase, aspartate aminotransferase, blood urea, blood lactate dehydrogenase, blood bicarbonate, decreased blood albumin, blood chloride, protein total, blood bicarbonate, blood bilirubin, hyperglycemia, hypoalbuminemia, hypomagnesemia, hypokalemia, hyponatremia, decreased appetite, anorexia, hyperkalemia, dehydration, hyperbilirubinemia, transfusion reactions, blurred vision, dysuria, urinary frequency.

However, if the aforementioned data indicate that both 5-Aza and decitabine have a tangible toxicity on normal tissues, recent biological data seem to suggest that normal cells may interact differently with DNMT inhibitors than malignant cells. In this regard, some data suggest that normal cells, dividing at a slower rate than malignant cells, incorporate less drug than cancer cells into their DNA resulting in decreased effect on DNA methylation. Zebularine, a novel DNA methyltransferase inhibitor, has properties of acting differentially on cancerous and normal cells. Continuous treatment with this drug substantially reduces the growth rate of human cancer cells with less effect on normal human fibroblasts. Growth inhibition in cancer cells was found to be associated with the induction of *p21 *which was unmodified in human fibroblasts. This suggests that the growth-suppressive potential of zebularine in tumor cells is seemingly *p21*-dependent and its differential effects was partially due to preferential incorporation of zebularine into DNA of tumor cells as documented by the uridine/cytidine kinase activity levels that were generally higher in cancerous than normal cells. Therefore, the preferential effects of zebularine in cancer cells in terms of incorporation into DNA, growth inhibition, demethylation, and depletion of DNMTs are probably due to differential metabolism compared to normal cells.

If different metabolism between normal and malignant cells may partially explain the preferential effect of DNMT inhibitors on tumor cells [167], the differential genes expression that these inhibitors induce in different cellular sub-populations is equally relevant. *Karpf *and coworker conducted a genomic analysis of gene expression modifications upon decitabine treatment both in normal and cancerous cell lines. In their analysis the authors concluded that decitabine (i) elicited changes in a limited number of genes, (ii) regulated gene expression similarly between normal and cancer cells, and (iii) changes in the expression of specific genes required the presence of transcriptional activators competent for activation of the target promoter [168,169]. However, if the differences in gene expression patterns between normal and malignant cells upon DNMT inhibitors seem to be less marked than previously seen, the selective activation of specific genes in tumor cells is clearly documented in literature. In this regard, decitabine leads to the selective activation of specific genes only in tumor cells opening to the intriguing hypothesis that DNMT inhibitors may increase the therapeutic index of specific antitumor strategies consenting of targeting the gene products differentially expressed in tumor cells [169].

Xerostomia is a common complication of radiotherapy on head and neck cancer due to irreparable damage caused to the salivary glands if they are included in the radiation fields. This side effect is perceived negatively by many patients with a significant impact on their quality of life. This condition is partially due to the death of a significant number of glandular ductal cells, which if not replaced, result in a significant decrease in the amount of produced saliva. *Motegi *and coworkers examined the mechanism by which immortalized normal human salivary gland ductal cells acquire the ability to express Aquaporin 5 (AQP5) and secrete fluid in response to decitabine treatment [170]. AQP5 is a water channel protein and its role in the generation of saliva, tears and pulmonary secretion is well documented. These authors suggest that decitabine resulted in AQP5 gene expression in human salivary gland ductal cells (NS-SV-DC cells) affecting the water permeability by increasing the transepithelial net fluid secretion of surviving ductal cells. However, the improvement in the efficiency of transepithelial fluid secretion may be of limited value if the noxa patogena holds over the response of normal tissue is the production of fibrosis that results in to the complete loss of tissue function. Therefore, drugs capable of reducing the fibrinogenic potential of specific treatments are of value in clinical setting.

Some authors documented that DNA methylation exert epigenetic control over fribrinogenesis and wound healing. These events need a process of cell transdifferentiation of resident cells to stellate cells, a particular subtype of myofibroblast, which seems to be a common process in the majority of soft tissues. Myofibroblasts are highly profibrinogenic and produce proinflammatory mediators (IL-6, MCP-1, PFGF, TGFβ-1) resulting in the secretion of a large quantities of collagen I and III [171]. MeCP2 is a methyl-CpG-binding protein that has the potential to exert regulation over the expression of multiple genes via its interaction with methylated DNA. MeCP2 is a repressor of IkBα which is required for transdifferentiation and profibrinogenic activity of stellate cells. Another important mediator of transdifferentiation and fribrinogenesis is PPARγ whose transcriptional silencing activity is required for conversion of hepatic stellate cells to myofibroblast [172]. Forced expression of PPARγ in hepatic myofibroblast results in reversing the transdifferentiation and profibrinogenic potential of stellate cells with down-regulation of type I collagen, loss of proliferation, and reacquisition of their adipogenic characteristics. Experimental data suggest that MeCP2 is recruited to the IkBα promoter and decitabine treatment modulating epigenetically the expression these mediator exerts control over key fibrinogenic and inflammatory transcriptional regulators reducing greatly the fibrogenic potential of stellate cells [173].

Another significant property of DNMT inhibitors is their capability to act as antioxidants under specific biological setting. This property is very important, especially in conditions where an excess of free radicals results in tissue damage. EGCG is a major element of green tea with a documented activity as DNMT inhibitor. Conflicting data are available in literature on the effect of EGCG on normal and tumoral cells. In an immortalized normal breast epithelial cell line (MCF10A), EGCG induced growth arrest prior to the cell cycle restriction point, with elevated p21, hypophosphorylation of Rb, and decreased cyclin D1, suggesting that higher concentrations of EGCG may be toxic to normal mammary epithelial cells [174]. However, EGCG may have both antioxidant and prooxidative activities involved in redox cycling and quinone formation [175] and may induce oxidative stress *in vivo *[176,177]. Cysteine conjugates, indicative of reactive species development, have been detected after 200 and 400 mg/kg i.p. EGCG [178]. EGCG was also reported to be capable of inducing liver, kidney and gastrointestinal toxicity which seemed to be correlated with bioavailability of EGCG [176,179]. Different results were documented by *Yamamoto *and his group [180] since new mechanisms by which EGCG may act differentially in tumor and normal cells were identified. In humans, EGCG is rapidly absorbed through the oral mucosa and secreted back into the oral cavity by saliva, suggesting that salivary glandular cells may tolerate high concentrations of EGCG [181]. Accordingly, this evidence suggests that EGCG may differentially affect oxidative status and may act as either a ROS inducer or a ROS suppressor depending upon the cell type [182]. EGCG concentrations higher than plasma *C*max do not produce ROS in cells derived from the normal epidermis and oral cavity but rather protect these cells by decreasing ROS production. Mechanisms responsible for the differential effects could rely on distinctive signal pathways activated by EGCG in a tissue-specific manner. High concentration of EGCG failed to produce ROS in normal epidermal keratinocytes, and immortalized normal salivary gland cells. In contrast, EGCG elevated ROS levels upon treatment in a dose-dependent manner in oral carcinoma cells. The ROS levels were significantly higher in the tumor cell lines that possessed low catalase activity. Therefore, EGCG may potentially simultaneously enhance tumor cell death rate and protect normal cells from chemo/radiation-induced oxidative stress in tumors such as skin and head neck [182].

If uncertainty exists regarding the effect of DNMT inhibitors on normal tissues, scanty direct evidence exists regarding the biological effects these inhibitors have in combination with chemotherapy or ionizing radiation. Clinical data indicate that, when decitabine is administered in association with cisplatin in subjects with advanced squamous cell carcinoma of the cervix, a significant hematological toxicity is documented [183].

Recent study reported interesting evidence of biological interaction of a DNMT inhibitor with chemotherapy. In this report 5-Aza was combined with cisplatin in order to measure the improvement in the therapeutic index of these two drugs. Interestingly, 5-Aza prevented the nephrotoxicity related to cisplatin, which was further related with the lowering in the levels of BUN and creatinine in the murine model. The mechanism by which 5-Aza decreased the nephrotoxicity involved the reduction of the levels of mediators such as metallothioneins, which are able to induce oxidative stress or indirectly activate gene responsible for preventing oxidative stress. This represents the first evidence that an epigenetic treatment with a DNMT inhibitor reduces the toxicity related to chemotherapy [184]. A single study dealing with the combination of decitabine with ionizing radiation reports in a differentiated effect in terms of growth inhibition normal and cancerous cells [185]. When decitabine was combined with radiation no significant synergism in terms of growth inhibition was found on human fibroblasts. On contrary, irradiation alone resulted in a significant decrease in the proliferation of normal fibroblasts, suggesting that decitabine significantly modified cellular response to irradiation.

### Off-target effects of DNMT inhibitors and potential contribution to radiosensitization

The mechanisms by which DNMT inhibitors exert their effects on cells may be divided into those related to DNMT inhibitors and those not related to demethylation of DNA. The latter mechanisms may be defined as off-target effects and although observed at higher concentrations may substantially contribute to the antitumor properties of nucleoside analogues alone or in combination with chemotherapy or ionizing radiation. Numerous chemicals as well as radiation can lead to covalent protein-DNA adducts. 5-Aza, one of the most important nucleoside analogue, leads to protein-DNA adducts. 5-Aza is a cytidine analogue in which carbon-5 (C5) of the pyrimidine ring is replaced with nitrogen. Normally, a DNA cytosine-C5 methyltransferase (MTase) acts on cytosine residue in its recognition sequence by covalent binding to C6, and then transferring the methyl group from S-adenosylmethionine to C5; the covalent protein-DNA adduct is then reversed and the enzyme dissociates from the DNA [186]. 5-Aza substitution at target cytosine interferes with the reaction cycle and results in long-lived or irreversible MTase-DNA adduct. A major consequence of 5-Aza treatment is loss of cytosine MTase activity [186]. In mammalian cells, 5-Aza results in defective tRNA and rRNAs and inhibits protein synthesis [187]. Additionally, decitabine results in the induction of p53 DNA damage response, proposed to be dependent on formation of MTase adducts [188]. Covalent protein-DNA adducts or tightly bound proteins represent a major challenge to the DNA replication machinery. DNA replication forks can be blocked *in vivo *by replication termination complexes [189]. As previously described, scanty evidences indicate that nucleoside analogues may be efficient radiosensitizers. The cytotoxic mechanisms of nucleoside analogues may potentiate the effects of ionizing radiation for several theoretical reasons. First, as DNA synthesis inhibitors, nucleoside analogues have a potential to inhibit the repair of genomic damage induced by ionizing radiation. Second, because they are preferentially cytotoxic to proliferative cells, these analogues may decrease the number of tumor clonogens and thus slow down cell repopulation during fractionated radiotherapy. Tumor shrinkage induced by these compounds may improve tumor oxygenation and counter the detrimental effect of tumor hypoxia on radiation response. Third, a nucleoside analogue with DNA chain terminator property may, following their incorporation into the DNA repair patch, trigger an apoptotic response similar to that observed during the replication phase. Since DNA damage is induced in all phase of the cell cycle by radiation, this mechanism offers the prospect of extending the cytotoxicity of these analogues to non-S-phase cells. Due to the very low number of studies facing this topic the aforementioned mechanisms are all putative and specific experimental studies *in vitro *and *in vivo *settings should be performed in order to confirm these hypotheses.

### Combination of DNMT inhibitors with HDAC inhibitors

Due to their relative antitumor selectivity DNMT inhibitors have been used in combination with HDAC inhibitors. Recently, there have been several excellent reviews of the histone deacetylase inhibitors (HDACIs) field used as a therapeutic option alone or in association with a variety of novel and conventional anticancer agents [190-192]. Optimal re-expression of methylated genes such as tumor suppressor or other cancer relevant genes following the serial application of a DNA methyltransferase inhibitor followed by an HDAC inhibitor created significant interest in combination epigenetic therapy. Among these p16INK4A and p14INK4b, Apaf-1 and caspase-8 are efficiently re-expressed when DNMT inhibitors and HDACIs are combined [190-192]. DNMT and a group of methyl-cytosine binding proteins, e.g., MeCP2, can also recruit and direct HDACs to the chromatin associated with silenced genes. Combined treatment with a DNMT inhibitor and HDACI has been shown to be superior in de-repressing silenced tumor suppressor genes, as well as in inducing increased growth inhibition, differentiation and apoptosis of cancerous cells. The best results in de-repressing silenced genes are observed when DNMT inhibitors are used first at relatively low doses followed by exposure to the HDACIs.

## Conclusions and Future Directions

No doubt exists that combining traditional cancer therapy with epigenetic modulators and reversing the changes of DNA methylation pattern holds a huge potential for successful treatment of haematological and solid malignancies. There are a number of important steps that need to be accomplished on the path towards efficient epigenetic therapy. Firstly, it is important to gain more insight into the diverse molecular mechanisms of the epi-drugs available today. A body of experimental evidences suggests that DNMT inhibitors may serve as efficient chemo- and radiosensitizers in solid tumors. However, the observation reported need more support in order to indicate intimate molecular mechanisms of chemo- and radiosensitization. Additionally, the understanding of different mechanisms as well as the long term safety of DNMT inhibitors on normal cells alone or in combination with standard treatments remains very limited and requires further research efforts. Although clinical and preclinical data indicate a substantial toxicity of these inhibitors, other evidence seems to suggest that these drugs may have potential in reducing toxicity under specific conditions. Therefore, the future goal is to indentify compounds able to enhance the therapeutic index and protect the non malignant tissues from side effects. Finally, other preclinical data suggest that the sensitizing effects of DNMT inhibitors seem to depend on the epigenetic modulation of a wide array of genes. A non negligible part of this effect may be related to the off-target mechanisms. Future research will focus on establishing clinically relevant combinations of DNMT inhibitors and conventional cancer therapies. In particular, a longstanding interest exists in the development of molecules that can modify cellular responses to radiation and chemotherapy. The future perspectives lie in identifying more similar compounds and elucidating their mechanisms of action in order to develop more effective cancer therapies and treatments.

## Competing interests

The authors declare that they have no competing interests.

## Authors' contributions

GLG, CF, FM, RGP and VT drafted and wrote the manuscript. VP and BMZ revised the manuscript critically for important and intellectual content. GLG and FM supervised the project. All authors read and approved the final manuscript.
